# *Bunium persicum* essential oil reduced acetic acid-induced rat colitis through suppression of NF-κB pathway

**DOI:** 10.22038/AJP.2021.18037

**Published:** 2021

**Authors:** Amir Rashidian, Dorna Akbarzadeh, Jinous Asgarpanah, Ahmadreza Dehpour

**Affiliations:** 1 *Department of Pharmacology, School of Medicine, Tehran University of Medical Sciences, Tehran, Iran*; 2 *Experimental Medicine Research Center, Tehran University of Medical Sciences, Tehran, Iran*; 3 *Department of Pharmacognosy, Faculty of Pharmacy and Pharmaceutical Sciences, Tehran Medical Sciences, Islamic Azad University, Tehran, Iran*

**Keywords:** Bunium persicum essential oil, Ulcerative colitis, Acetic acid, NF-kB

## Abstract

**Objective:**

The aim of this study was to evaluate the anti-inflammatory effect of *B. persicum *essential oil on colonic inflammation and the role of suppression of NF-κB pathway in rat colitis induced by acetic acid solution.

**Materials and Methods:**

Induction of acute colitis was done by intra-luminal instillation of 2 ml of acetic acid (4%) diluted in normal saline. Two hours after colitis induction, 0.2% tween 80 in normal saline, prednisolone (4 mg/kg) or* B. persicum* essential oil (100, 200, and 400 mg/kg) were administered to the rats orally and continued for 5 consecutive days. The severity of macroscopic and microscopic damages was assessed. Myeloperoxidase and TNF-α activity was evaluated by biochemical analysis and ELISA respectively and protein expression of p-NF-κB was assessed by immunohistochemistry (IHC).

**Results:**

Prednisolone and *B. persicum* essential oil (100, 200, and 400 mg/kg) decreased macroscopic and microscopic injuries compared to the acetic acid group. On the other hand, prednisolone and* B. persicum* essential oil (200 and 400 mg/kg) decreased the activity of MPO and TNF-α in the colon tissue of rats compared with the acetic acid group. Furthermore, they suppressed the expression of p-NF-κB protein induced by acetic acid administration.

**Conclusion:**

It is suggested that the anti-inflammatory effect of *B. persicum* essential oil on acetic acid-induced colitis in rats may be due to the suppression of NF-κB pathway.

## Introduction

Inflammatory bowel disease (IBD) including ulcerative colitis (UC) and Crohn’s disease (CD), is an immune-mediated inflammation of the gastrointestinal (GI) tract. The sign and symptom of IBD include bloody diarrhea, fever, abdominal pain and cramping and reduction of appetite (Park et al., 2017; Dejban et al., 2020). There are several factors that affect etiopathogenesis of the disease such as genetics, environment, infectious agents and dysregulation of the immune system. In other words, interaction between these factors induce inflammatory responses of intestinal mucosa that result in initiation and progression of IBD (Ananthakrishnan et al., 2018). Short and long-term treatments of IBD with current drugs such as glucocorticoids and aminosalicylates have low efficacy and high adverse effects including osteoporosis and hematological disorders (Curkovic et al., 2013). Complementary and alternative medicines have been used for centuries by peoples for the treatment of different inflammatory conditions because of their good efficacy and low side effects. Many reports have shown that plants contain different medicinal compounds with anti-inflammatory effects (Chamanara et al., 2019; Rezayat et al., 2018, Goudarzi et al, 2020). Essential oils are volatile chemical compositions extracted from plants by hydrodistillation. They are used for the treatment of inflammatory conditions as an alternative medicine (Baptista-Silva et al., 2020). *Bunium persicum* (Bioss) popularly known in Persian as “Zireh Kohi” belongs to the family of Apiaceae that grows widely in the southeast regions of Iran (Moradi Mehrabadi and Zarshenas, 2020). The plant seeds have been traditionally used as a flavoring agent, carminative, anti-spasmodic, antiepileptic and stimulant of milk secretion (Zargari, 1996). Several studies have reported antinociceptive, antioxidant, anti-inflammatory, antimicrobial and antitoxoplasmosis properties of the essential oil of *B. persicum* (Hajhashemi et al., 2011; Kareshk et al., 2015; Sekine et al., 2007; Talei et al., 2009). Based on the above mentioned information, the aim of this study was to evaluate anti-inflammatory effect of *B. persicum* essential oil on acetic acid-induced colitis in rats. 

## Materials and Methods

**Chemicals**

Prednisolone, O-dianisidine dihydrochloride and hexadecyl trimethyl-ammonium bromide (HTAB) were obtained from Sigma Chemical Co (St Louis, Minneapolis). Formalin solution 35% w/w and glacial acetic acid were purchased form Merck (Darmstadt, Germany). Also, 0.2% Tween 80 in saline solution was used as a vehicle for preparing suspension of prednisolone and emulsion of *B. persicum* essential oil. 

**Plant and essential oil preparation**

*Bunium persiucm* seeds were obtained from the medicinal plant institute, Shahid Beheshti University, Tehran, Iran. A voucher specimen (No. MPH-1398) was determined by Dr Asgarpanah at the Herbarium of Faculty of Pharmacy, Pharmaceutical Sciences Branch, Islamic Azad University (IAUPS), Tehran, Iran. The essential oil was extracted from the air-dried, powdered plant seeds by hydrodistillation for 3 hr, based on the method recommended in European pharmacopoeia 23 (Council of Europe, 2002).

**Analysis of the essential oil**

Gas chromatography‒mass spectroscopy (GC‐MS) was carried out by using an HP‐6890 gas chromatograph (GC) coupled with an HP‐5973 Mass Selective Detector (MSD) (Agilent Technologies, Santa Clara, CA) functioning at 70 eV mode. Chemicals presents in the essential oil were identified by comparing their mass spectra with those deposited in the mass spectral libraries, including Wiley NBS75K.L and NIST/EPA/NIH (2002 version; National Institute of Standards and Technology, Gaithersburg, MD), using different search engines. The relative percentage of the oil constituents was calculated by using Peak areas (Contini et al, 2020). 

**Animals**

Wistar rats weighing 200±20 g were provided from Pharmacology Department, School of Medicine, Tehran University of Medical Sciences, Tehran, Iran. Animals were kept in cages (at 20–23^o^C, with 50–60% humidity, and 12 hr/12 hr dark/light cycle) with free access to standard animal feed and water *ad libitum*. All experiments were performed in accordance with ‘‘Principles of Laboratory Animal Care’’ (NIH publication 82-23, revised in 1985 and further implemented in 1996) and approved by the ethical committee of Tehran University of Medical Sciences, number 65-76-03, 01 December 2019.

**Colitis induction**

Induction of colitis was done based on our previous studies (Goudarzi, Partoazar et al. 2020, Rashidian, Keshavarz-Bahaghighat et al. 2019). Rats were fasted for 24 hr before colitis induction with free access to water. Animals were anesthetized using ketamine (100 mg/kg) and xylazine (10 mg/kg). Then, diluted solution of acetic acid 4% (2 ml) was intra-rectally instilled through a flexible plastic catheter with the outside diameter (2 mm) inserted 8 cm into the rectum.

**Experimental plan**

Rats were randomly selected into six groups, each group included six rats: Control group: without colitis induction+0.2% tween 80 in normal saline/day; Acetic acid group: 2 ml acetic acid 4%+0.2% tween 80 in normal saline/day; Prednisolone group: 2 ml acetic acid 4%+prednisolone 4 mg/kg/day; Essential oil 100 group: 2 ml acetic acid 4% +essential oil 100 mg/kg/day; Essential oil 200 group: 2 ml acetic acid 4%+essential oil 200 mg/kg/day; and Essential oil 400 group: 2 ml acetic acid 4%+essential oil 400 mg/kg/day (Hajhashemi, et al. 2011). Colitis induction was carried out on the first day and treatments were started 2 hr after induction and followed for 5 consecutive days (Rezayat, et al. 2018). Oral gavage was used as a method of administration to the animals. Rats were killed 24 hr after the last treatment (day 6) in a CO_2_ chamber and the last 8 cm of the colon from the anus was excised.

**Evaluation of macroscopic injury**

Colon tissue of each rat was opened lengthwise and cleaned with normal saline. The ulcer index was used for evaluating colonic damage. This index was calculated based on this formula: Ulcer index: Ulcer area (cm^2^) + Ulcer severity. Ulcer area was calculated by using 3 M® surgical transparent tape for measuring the damage area of the colon. Ulcer severity was measured based on this scale: 0=no macroscopic change, 1=mucosal erythema only, 2=mild mucosal edema, slight bleeding, or slight erosion, 3=moderate edema, bleeding ulcers or erosions and 4=severe ulceration, erosions, edema, and tissue necrosis (Rashidian et al., 2014).

**Evaluation of histological injury**

These methods were used to prepare the colon tissue: fixation in 10% formalin, dehydration, embedding in paraffin wax, processing, cutting into 4 µm-thick sections, and staining with hematoxylin and eosin (HE). A histopathologist, blinded to the treatment, assessed colon tissues based on the scale of 0-5 (Table 1). 

**Immunohistochemical analysis of pNF-kB**

Phosphorylated NF-kB expression was assessed by immunohistochemistry method. Here, 4 µm of colon slices was embedded in paraffin, deparaffinized in xylene, washed with alcohol, and finally rehydrated by phosphate buffered saline (PBS). Retrieval of antigen was performed in citrate buffer (pH 6.0) then incubated with blocking solution (3% Bovine serum albumin).

**Table 1:** Criteria of inflammation grading

Grades	Histological feature
Grade 0	Normal mucosa, submucosa, muscularis propria, and serosa without inflammatory cells
Grade 1	Inflammatory cells in mucosa and submucosa
Grade 2	Transmural acute inflammation
Grade 3	Small ulcers (up to 3) with acute inflammation in the wall
Grade 4	Multiple large ulcers with transmural inflammation
Grade 5	Extensive ulceration with necrosis of the entire wall or part of it, transmural inflammation, and irregular villous mucosal surface

The incubation of slides was done using mouse monoclonal IgG1 (kappa light chain) p-NF-κB p65 (27. Ser 536) (SC136548) Primary antibody (1:100 dilution), at 4°C overnight. Then, the slides were washed four times with 3% H_2_O_2_ at room temperature. Thereafter, the incubation process was done with mouse IgG kappa-binding protein (m-IgGκ BP) conjugated to horseradish peroxidase (HRP) (1:50 dilution) antibody (Santa Cruz Biotechnology, USA) for 1 hr After washing the slides with PBS, they were stained with diaminobenzidine solution (DAB; Boster Biological Technology, USA). After that, staining was stopped by distilled water and counterstaining was performed by hematoxylin. A histopathologist unaware to the treatment assessed colonic tissues by the previous scoring system (Allred, et al. 1998). Immunohistochemical scores were calculated based on the sum of a proportion score (percentage of positive stained cells as none = 0, below 1% = 1, 1–10% = 2, 11–33% = 3, 34–66% = 4, and 67–100% = 5) and an intensity score (none=0, weak=1, intermediate=2, and strong=3). The numerical score rating was as follows: negative=0–1, weak positive=2–3, positive=4–6, and strong positive=7–8.

**Evaluating of TNF-α activity**

The activity of TNF-α was evaluated by using rat TNF-α enzyme-linked immunosorbent assay (ELISA) kit according to the manufacturer’s instructions (CUSABIO Technology LLC., USA). The pieces of colonic tissues were homogenized in 50 mmol/L ice-cold potassium phosphate buffer (pH 6.0); then, homogenate was centrifuged at 4°C for 20 min. Then, supernatant was extracted and stored at − 80°C. TNF-α level is reported as picogram per milligram.

**Evaluating of MPO activity**

The assessment of myeloperoxidase (MPO) activity in the colon tissue, was carried out by the method formerly described (Bradley 1982). A 0.1-g section from the tissue was homogenized in 1 ml of potassium phosphate (pH 6) containing 0.5% HTAB. Then, the buffer was added to the final volume of 5 ml, and the homogenate was sonicated for 10 sec in an ice bath. Afterward, the homogenate was centrifuged at 15,000 rpm, at 4°C for 15 min. Then, 0.1 ml of the supernatant was diluted by phosphate buffer saline (50 mM, pH 6), containing 0.0005% hydrogen peroxide and 0.167 mg/ml odianisidine dihydrochloride to the final volume of 3 ml. Eventually, the change in the suspension absorption at 460 nm was measured by a spectrophotometer. MPO activity is reported as unit per gram. 

**Statistical analysis**

The data was analyzed by using GraphPad Prism (Ver.5.04). The differences among groups were identified by one-way analysis of variance (ANOVA) followed by Tukey’s *post hoc* test, and s p<0.05 was considered significant. All data are presented as mean±SEM.

## Results

**Essential oil analysis**

Eight compounds were characterized in the plant seeds essential oil by GC/MS analysis (Table 2). Among them, four compounds were considered to be the main compounds including p-cumin aldehyde (29.41%), cuminic alcohol (22.91%), γ-terpinene (12.98%) and carvacrol (12.13%).

**Table 2:** Constituents of the essential oil of *B. persicum*

No	Compound	Content (%)	Retention index
1	β-Pinene	4.22	974
2	p-Cymene	2.63	1021
3	γ-Terpinene	12.98	1057
4	p-Cumin aldehyde	29.41	1247
5	Cuminic alcohol	22.91	1289
6	3-Phenyl-2-butanol	12.05	1296
7	Carvacrol	12.13	1297
8	cis-Sabinyl acetate	0.42	1329

**Macroscopic results**

**Figure 1A:**
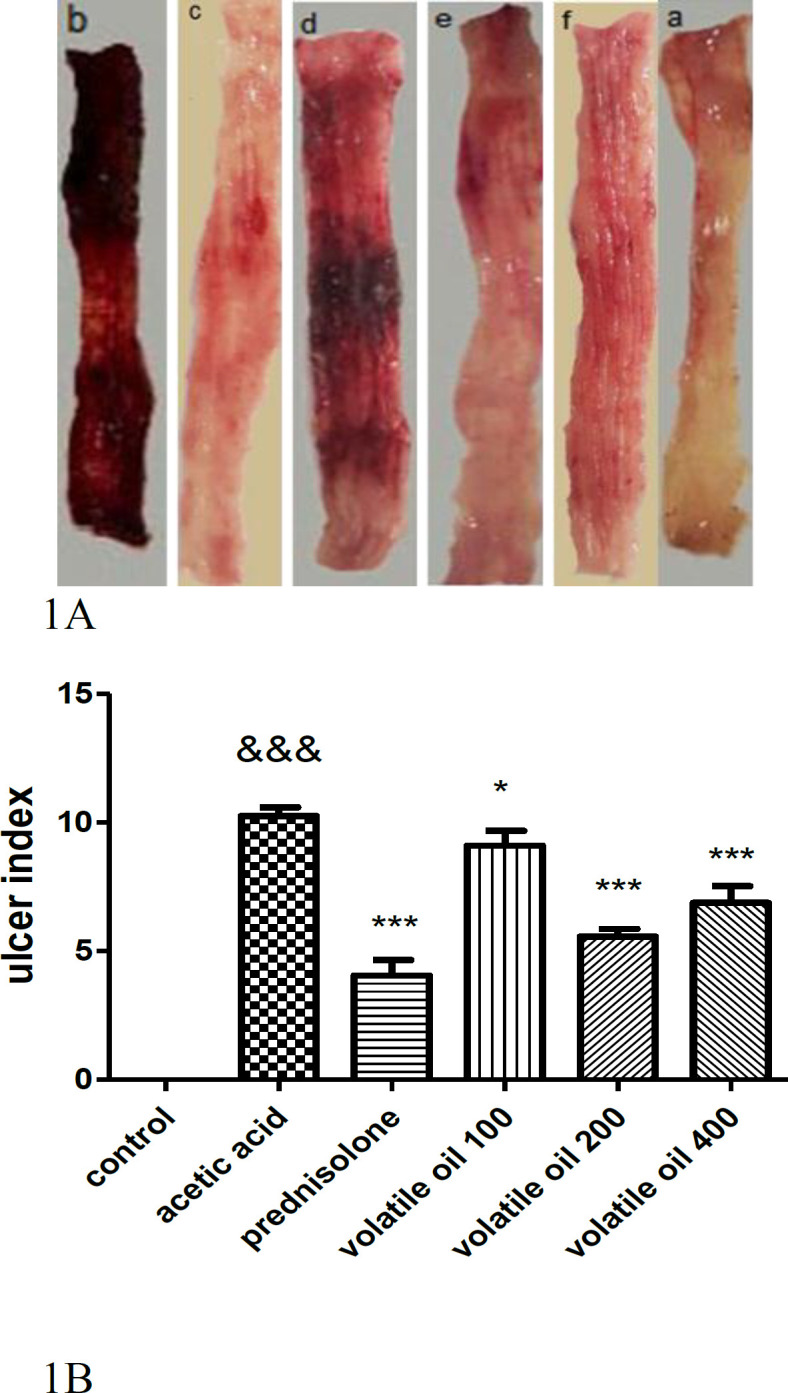
Images of colon tissue six days after induction of colitis by acetic acid. a, control; b, acetic acid; c, prednisolone (4 mg/kg) ; d-f; *B. persicum* essential oil 100, 200, and 400 mg/kg, respectively. 1B. Effect of prednisolone (4 mg/kg); d-f; *B. persicum* essential oil (100, 200, and 400 mg/kg) on ulcer index. Results are presented as mean±SEM; (n = 6 rats/group). ^&&&^p<0.001 compared to the control group, ***p<0.001 and *p<0.05 compared to the acetic acid group. The analysis was done by one-way analysis of variance (ANOVA) followed by Tukey’s *post hoc* test

None of the animals died after receiving 4% acetic acid (2 ml). After macroscopic evaluation of the colon, no epithelium injury was seen in the control group, but the acetic acid group showed severe mucosal damage along with necrosis. On the other hand, administration of prednisolone or *B. persicum* essential oil led to reduction of macroscopic injury compared to the acetic acid group (Figure 1A). As shown in Figure 1B, induction of colitis led to an increase in ulcer index (summation of ulcer area and ulcer severity) compared to the control group (p<0.001). In contrast, administration of prednisolone (4 mg/kg) or *B. persicum* essential oil (100, 200 and 400 mg/kg) reduced ulcer index compared with the acetic acid group (p<0.05, p<0.001). 

**Histopathological results**

**Figure 2:**
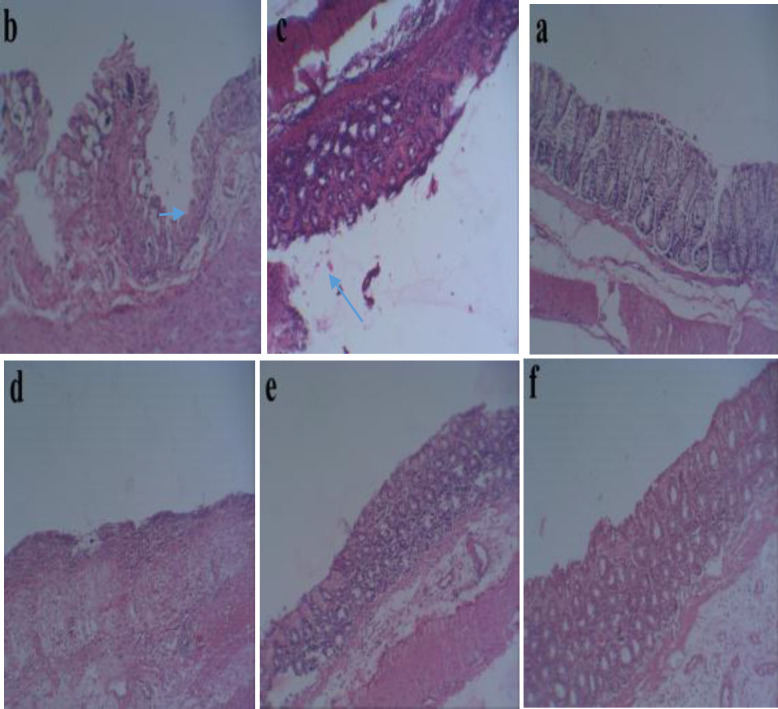
Pathological characteristics of the colon tissue of rats. Representative hematoxylin and eosin stained of colon sections. Magnifications: ×10. a, control; b, acetic acid; c, prednisolone (4 mg/kg); d-f; *B. persicum* essential oil (100, 200, and 400 mg/kg), respectively

The colon histopathological damage was graded on a scale of zero to five as shown in Table 1. As shown in Figure 2, the control group revealed normal mucosa, submucosal, muscularis propria and serosa without inflammation and necrosis. On the other hand, induction of colitis resulted in damage to the epithelium, extensive ulceration with necrosis of the entire wall or part of it and inflammatory cells infiltration such as neutrophils. In contrast, administration of prednisolone (4 mg/kg) and *B. persicum* essential oil (200 and 400 mg/kg) reduced epithelium damage, necrosis and infiltration of neutrophils. Moreover, *B. persicum* essential oil (100 mg/kg) administration did not reduce histopathological damage compared to the acetic acid group.

**Immunohistochemical results**

**Figure 3:**
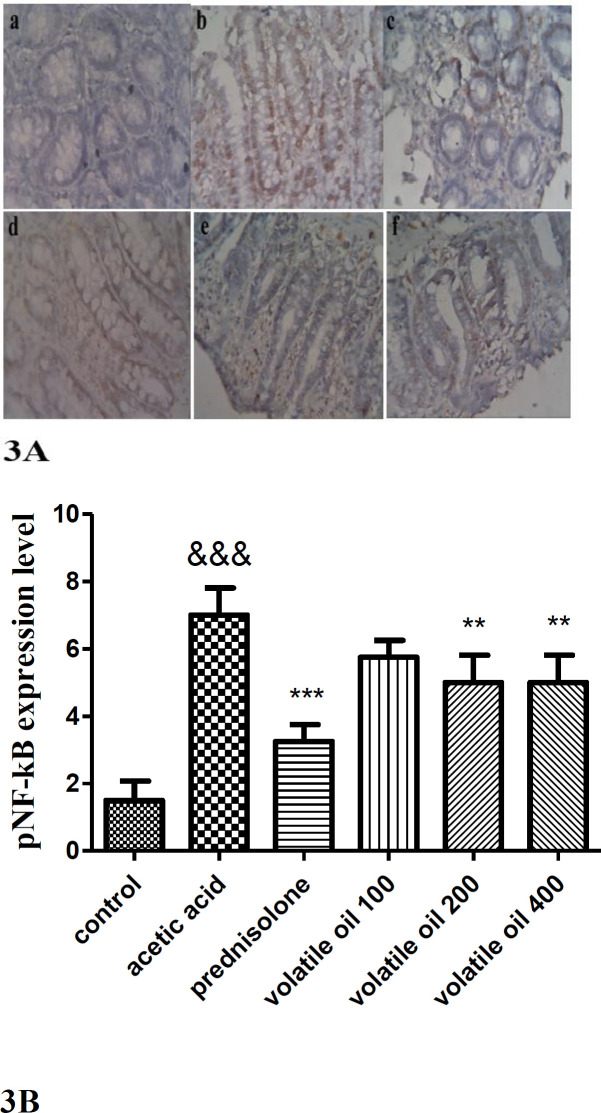
Immunohistochemical analysis of pNF-κB expression in the colon tissue of rats. A Immunohistochemical staining of colon tissue sections for pNF-κB (×40). a, control; b, acetic acid; c prednisolone (4 mg/kg); d-f; *B. persicum* essential oil (100, 200, and 400 mg/kg), respectively. B pNF-κB positive cells (brown colors) expression in the colon tissue. Results are means±SEM; (n=6 rats/group). &&&p<0.001 compared to the control group, **p<0.01 compared to the acetic acid group. The statistical analysis was done by one-way analysis of variance (ANOVA) followed by Tukey’s *post hoc* test

Figure 3A and B revealed immunohistochemical staining of pNF-κB. As it is shown, the expression of pNF-κB positive cells (brown colors) were not observed in the colon tissue of the control group. But, induction of colitis by acetic acid led to an increase in the expression of pNF-κB positive cells (brown colors) compared to the control group (p<0.001). On the other hand, administration of prednisolone (4 mg/kg) or *B. persicum *essential oil (200 and 400 mg/kg) reduced the expression of pNF-κB positive cells (brown colors) significantly (p<0.001, p<0.01, respectively). In addition, administration of *B. persicum* essential oil (100 mg/kg) did not significantly reduce the increased expression of pNF-κB positive cells (brown colors) compared with the acetic acid group (p>0.05). 

**TNF-α assessment results**

**Figure 4:**
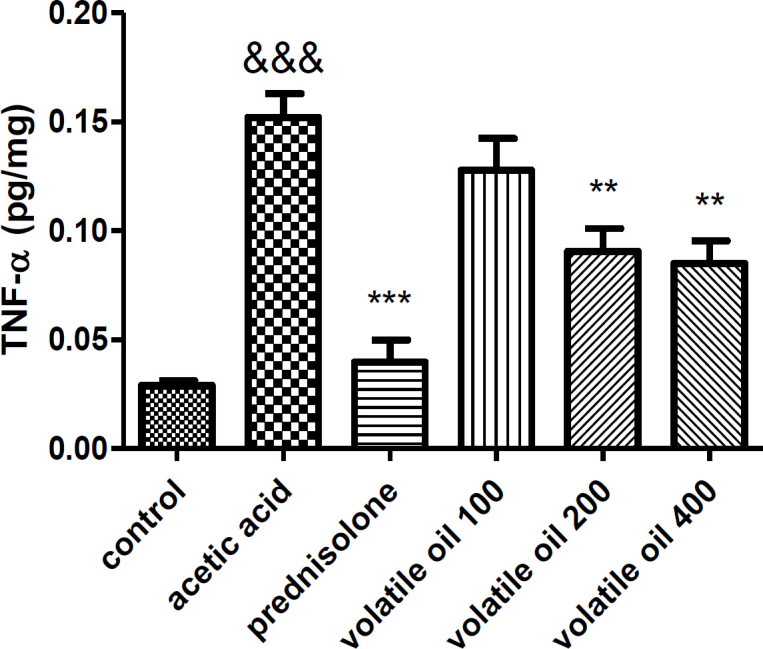
ELISA measurement of Tumor necrosis factor-α (TNF-α) activity in the colon tissue of rats. Results are expressed as mean±SEM; (n=6 rats/group). &&&p<0.001 compared to the control group, ***p<0.001 and **p<0.01 compared to the acetic acid group. The statistical analysis was done by one-way analysis of variance (ANOVA) followed by Tukey’s *post hoc* test

As it is depicted in Figure 4, the level of TNF-α was low in the control group. Induction of colitis by acetic acid caused significant increases in the level of TNF-α compared to the control group (p<0.001). On the other hand, administration of prednisolone (4 mg/kg) and *B. persicum *essential oil (200 and 400 mg/kg) significantly decreased the level of TNF-α compared with the acetic acid group (p<00.01 and p<0.01 respectively). In addition, administration of *B. persicum *essential oil (100 mg/kg) did not significantly decrease the increased level of TNF-α compared with the acetic acid group (p>0.05).

**Myeloperoxidase (MPO) assessment results**

**Figure 5:**
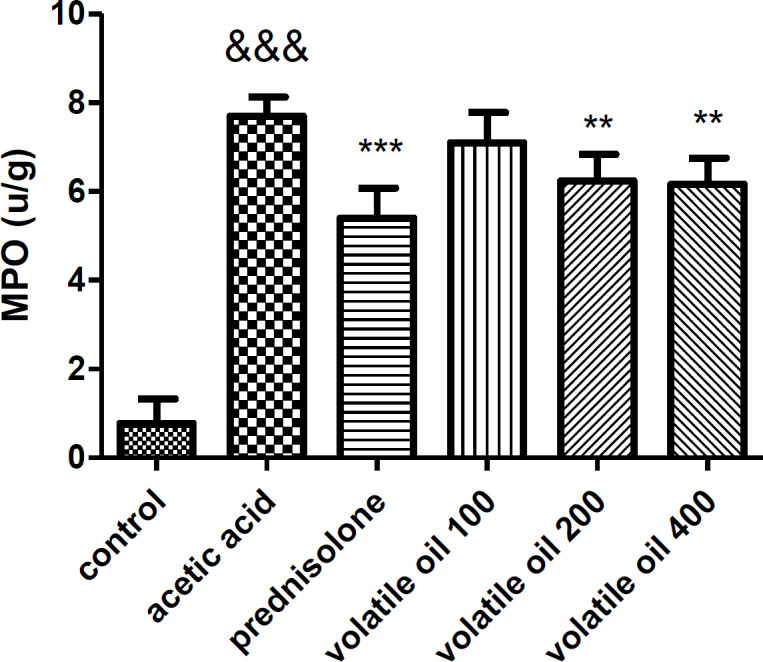
Effect of *B. persicum* essential oil (100, 200, and 400 mg/kg) and prednisolone (4 mg/kg) on MPO activity in the colon tissue of rats. Results are expressed as mean±SEM; (n=6 rats/group). ^&&&^p<0.001 compared to the control group, ***p<0.001 and **p<0.01 compared to the acetic acid group. The statistical analysis was done by one-way analysis of variance (ANOVA) followed by Tukey’s *post hoc* test

As shown in Figure 5, induction of colitis by acetic acid led to significant increases in the activity of MPO enzyme compared to the control group (p<0.001). In contrast, animals treated with prednisolone (4 mg/kg|) or* B. persicum* essential oil (200 and 400 mg/kg) decreased the activity of MPO in the colon tissue of rats compared with the acetic acid group (p<00.01 and p<0.01 respectively). In addition, *B. persicum* essential oil (100 mg/kg) did not significantly reduce the activity of MPO enzyme compared to the acetic acid group (p>0.05). 

## Discussion

This study showed that *Bunium persicum* essential oil reversed colonic damage induced by acetic acid in a rat model of colitis through reduction of macroscopic lesion, microscopic injury and MPO enzyme activity as well as the level of inflammatory mediator TNF-α and overexpression of nuclear transcription factor NF-κB. 

Also, the results of this study are in accordance with the previous evaluation on the anti-inflammatory properties of *B. persicum* essential oil against carrageenan-induced rat paw edema and croton oil-induced ear edema (Hajhashemi et al., 2011). Acetic acid is one of the important chemicals used for induction of colitis in laboratory animals such as rats. Intracolonic instillation of acetic acid caused mucosal ulceration, necrosis, crypt destruction, edema and transmural inflammation in the colon tissue (Chamanara et al., 2019). In the current study, intraluminal administration of acetic acid led to destruction epithelial layer, necrosis and inflammatory cells infiltration such as neutrophils. Instead, *B. persicum* essential oil decreased macroscopic lesions and histological signs of inflammation such as edema, transmural inflammation and necrosis.

The nuclear transcription factor kappaB (NF-κB) is one of the most important regulators in the immunological responses of colon mucosa. After colitis induction, NF-κB is activated and induces the expression of some pro-inflammatory genes results in the production of various cytokines (Dou et al., 2013). It has been shown that some constituents such as NF-κB blockers (corticosteroids) suppress NF-κB signaling pathway results in the inhibition of inflammatory cascades (Du et al., 2017; Fu et al., 2017). This study showed that administration of *B. persicum* essential oil led to a significant reduction of NF-kB expression in the colon tissue of acetic acid induced colitis in animals.

Tumor necrosis factor-α (TNF-α) is one of the most important pro-inflammatory mediators that plays a key role in the development and progression of IBD. In addition, it has been shown that there is a genetic association between high levels of TNF-α and IBD severity (Sands and Kaplan, 2007). Recent studies have revealed that blocking of TNF-α activity results in the reduction of inflammation in the colon in both experimental colitis model and patients with ulcerative colitis (Lv et al., 2014; Rashidian et al., 2019). This study showed that *B. persicum* essential oil significantly reduced the high level of TNF-α in the colon tissue of acetic acid-induced colitis.

Myeloperoxidase (MPO) is a proteolytic enzyme with a molecular weight of 140-kDa that exists in neutrophil granulocytes. After colitis induction, the activated neutrophils release this enzyme which results in the interaction between hydrogen peroxide and chloride ions and hypochlorous acid formation ultimately leading to the colon oxidative damage (Kannan and Guruvayoorappan, 2013; Prabhu and Guruvayoorappan, 2014). Measurement of MPO enzyme plays an important role in identifying the extent of damage to colon tissue (Klebanoff et al., 2013). The results of this study revealed that administration of *B. persicum* essential oil reduced the activity of MPO enzyme in the colon tissue after induction of colitis with acetic acid.

Phytochemical screening of *B. persicum* essential oil indicated different constituents such as p-cuminaldehyde, Cuminic alcohol, γ-terpinene, β-pinene, p-cymene and carvacrol. Cuminaldehyde one of the active constituents existing in the essential oil has revealed anti-inflammatory effect through inhibition of mRNA expression of proinflammatory cytokines such as interleukin 1 and 6, inducible nitric oxide synthase and cyclooxygenase type II. In addition, cuminaldehyde reduced protein levels of NF-κB, phosphorylated extracellular signal regulated kinase and phosphorylated c-Jun N-terminal Kinase in lipopolysaccharide-induced inflammation in cultured macrophage cells (Curkovic et al., 2013; Ebada, 2017). Furthermore, it has been shown that γ-terpinene reduced paw edema induced by agents such as carrageenan, prostaglandin-E2, histamine, or bradykinin. In addition, γ-terpinene decreased neutrophil migration as well as production of interleukin-1β and TNF-α in carrageenan-induced peritonitis in mice (de Oliveira Ramalho et al., 2015). Bonjardim et al have shown anti-inflammatory effect of p-cymene that was mediated through reduction of leukocyte migration in carrageenan-induced mice paw edema (Bonjardim et al., 2012). Lima et al have shown anti-inflammatory effect of carvacrol in the complete Freund’s adjuvant (CFA)-induced paw inflammation in mice that was mediated through decreasing the level of IL-1β and PGE2 and increasing the level of IL-10 (da Silva Lima et al., 2013). 

In conclusion, this study displays that *B. persicum* essential oil reduced acetic acid-induced colitis in rats that was mediated through suppression of NF-κB signaling pathway. This anti-inflammatory activity may be because of the presence of some constituents such as cuminaldehyde, γ-terpinene, p-cymene and carvacrol, which are known to have anti-inflammatory activity. Nevertheless, it is needed to assess more about the molecular mechanism of *B. persicum* essential oil and its isolated compounds as a supplement in the treatment of IBD.

## Conflicts of interest

The authors have declared that there is no conflict of interest.
